# A Delayed Morning and Earlier Evening Time-Restricted Feeding Protocol for Improving Glycemic Control and Dietary Adherence in Men with Overweight/Obesity: A Randomized Controlled Trial

**DOI:** 10.3390/nu12020505

**Published:** 2020-02-17

**Authors:** Evelyn B. Parr, Brooke L. Devlin, Bridget E. Radford, John A. Hawley

**Affiliations:** 1 Exercise and Nutrition Research Program, Mary MacKillop Institute for Health Research, Australian Catholic University, Melbourne 3000, Australia; b.devlin@latrobe.edu.au (B.L.D.); bridget.radford@acu.edu.au (B.E.R.); john.hawley@acu.edu.au (J.A.H.); 2 Department of Dietetics, Nutrition and Sport, La Trobe University, Melbourne 3086, Australia

**Keywords:** glucose, insulin, diet, obesity, appetite, acceptability, fasting, dietary patterns, metabolism

## Abstract

We determined the effects of time-restricted feeding (TRF; 8 h/d) versus extended feeding (EXF; 15 h/d) on 24-h and postprandial metabolism and subjective opinions of TRF in men with overweight/obesity. In a randomized crossover design, 11 sedentary males (age 38 ± 5 y; BMI: 32.2 ± 2.0 kg/m^2^) completed two isoenergetic diet protocols for 5 days, consuming meals at 1000, 1300 and 1700 h (TRF) or 0700, 1400 and 2100 h (EXF). On Day 5, participants remained in the laboratory for 24 h, and blood samples were collected at hourly (0700–2300 h) then 2-hourly (2300–0700 h) intervals for concentrations of glucose, insulin and appetite/incretin hormones. Structured qualitative interviews were conducted following completion of both dietary conditions and investigated thematically. Total 24-h area under the curve (AUC_total_) [glucose] tended to be lower for TRF versus EXF (−5.5 ± 9.0 mmol/L/h, *p* = 0.09). Nocturnal glucose AUC was lower in TRF (−4.2 ± 5.8 mmol/L/h, *p* = 0.04), with no difference in waking glucose AUC or AUC_total_ for [insulin]. Attitudes towards TRF were positive with improved feelings of well-being. Barriers to TRF were work schedules, family commitments and social events. Compared to extended feeding, short-term TRF improved nocturnal glycemic control and was positively perceived in men with overweight/obesity.

## 1. Introduction

The prevalence of overweight/obesity and type 2 diabetes mellitus (T2DM) continues to rise. Indeed, ‘Diabesity’ (the co-existence of obesity and T2DM) has been predicted to become the biggest epidemic in human history [1]. The proliferation in the rate of diagnosis of T2DM and other associated metabolic disorders is exacerbated in industrialized nations [2] where excess energy consumption and irregular eating patterns are prevalent [3]. To date, dietary strategies for the treatment and prevention of overweight, obesity and T2DM have focused on reducing energy intake and improving diet quality [4]. However, long-term adherence has been met with limited success [5,6] and current dietary guidelines do not take into consideration the *timing* of daily food intake. 

Irregular daily eating patterns have been shown to have adverse effects on circadian biology [7], independent of meal size and macronutrient composition [8,9]. The current 21st century lifestyle, encompassing around-the-clock eating behaviors [10], reduces the duration of time spent fasting such that most individuals are in a persistent postprandial state [11]. Recently, dietary strategies that focus on the timing of eating and duration of fasting (i.e., chrono-nutrition), rather than the type, quality or quantity of foods, have been demonstrated to improve metabolic health independent of weight loss [12,13]. Specifically, time-restricted feeding (TRF, in which daily food intake is restricted to between 8–10 h) is a dietary strategy that has emerged as a practical intervention for weight loss, and improving insulin resistance along with other markers of whole-body health [10,12,13,14,15,16]. Controlled (i.e., meals provided) ‘early’ TRF (eTRF; 0800–1500 h) improved both beta cell function in men with prediabetes [13] and 24-h glucose area under the curve (AUC) in individuals with obesity [12]. However, in those investigations, individuals were required to cease eating by 1500 h, potentially limiting the applicability and acceptance of such an extreme TRF approach. To date, only one study has compared the time of day of the TRF window (i.e., early (0800–1700 h) versus delayed (1200–2000 h)) on glucose tolerance [17]. The metabolic consequences of delaying the first meal to mid-morning to align with a more feasible 8-h TRF period of meal consumption have not yet been investigated.

Long-term adherence to dietary modifications for improving metabolic health is more burdensome than taking medication for people with T2DM [18]. Therefore, dietary interventions that are simple, practical and effective are urgently required to reduce the burden at both the individual and population level. To date, there are no published reports on the attitudes, opinions and perceived barriers of individuals who have participated in a short-term, controlled TRF intervention, which is required for future application of TRF as a dietary strategy. Therefore, we explored the metabolic effects and the subsequent attitudes and opinions of a short-term TRF protocol in which the first energy intake of the day was delayed (i.e., eating between 1000 and 1800 h) versus an isoenergetic extended feeding intervention (EXF; 0700 to 2200 h) in men with overweight/obesity. We hypothesized that a delayed-breakfast TRF dietary intake pattern would improve 24 h and postprandial glycemic control, compared to an EXF pattern, following the consumption of diets that were matched for energy and macronutrient composition.

## 2. Materials and Methods 

### 2.1. Study Design

**Figure 1:**
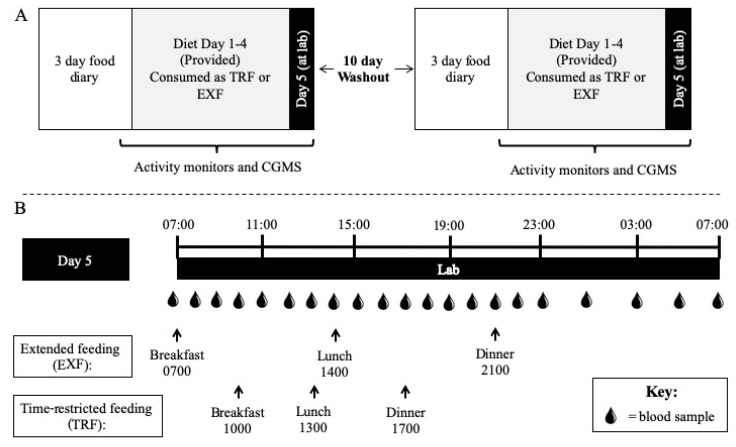
Study design schematic. Participants visited the laboratory at Day 0 to have activity monitors attached, continuous glucose monitoring system (CGMS) inserted and pick up Days 1–4 of the diet (50% total energy intake (TEI) fat, 30% TEI carbohydrate and 20% TEI protein) to be consumed in a time-restricted feeding (TRF) (1000, 1300 and 1700 h) or extended feeding (EXF; 0700, 1400 and 2100 h) pattern, in a randomized order separated by a 10 day washout period (**A**), before returning at 0645 h on Day 5 for a 24-h laboratory trial (**B**).

Each participant completed two experimental trials in a randomized, crossover design, with a 10-day wash out period between trials (Figure 1), at the St Patrick’s (Fitzroy, Victoria) campus of Australian Catholic University (ACU; February–July 2017). The study was approved by the ACU Human Research Ethics Committee (2016-215H), retrospectively registered (31/01/2017; first enrolment 30/01/2017, trial completion 22/06/2017) with the Australian New Zealand Clinical Trials Registry (ACTRN12617000165381) and eligible participants provided informed consent to attend the laboratory on five separate occasions (i.e., baseline, pre-condition 1, condition 1, pre-condition 2, condition 2). To assess an individual’s circadian preference, participants were asked to complete a morning-eveningness questionnaire self-assessment (MEQ-SA) [19,20].

### 2.2. Participants 

Men (aged 30–45 y) with overweight/obesity (body mass index (BMI) 27.0–35.0 kg/m^2^) and an inactive/sedentary lifestyle (<150 min/week of moderate-intensity exercise for >3 months and >3 h/day sitting) were recruited through flyers and social media. Potential participants were excluded if they met any of the following exclusion criteria: major or chronic illness that impaired mobility or eating/digestion (i.e., type 2 diabetes, etc.); previous bariatric surgery; shift workers; smokers (including e-cigarettes); individuals with strict dietary intake regimes (i.e., vegan, avoidances of principal study foods); individuals who did not regularly consume a breakfast meal or who did not have a regular consumption of three meals per day (i.e., breakfast, lunch and dinner); individuals who were currently restricting their dietary intake (i.e., activity trying to lose weight); individuals who were not weight stable (defined as <5 kg weight change) for the previous 3 months; and those who had undertaken international travel across several time zones <3 months before the trial commencement. Potential participants were also excluded if their self-reported wake and sleep times differed by >2 h across a week. Participants who were on any medications for sleep or glucose control were also excluded. No glycemic phenotyping measures (i.e., oral glucose tolerance or clamp tests) was performed in this cohort. Telephone pre-screening comprised of medical history, age, height and body mass (BM; to calculate BMI), weight history and screening of dietary habits. 

Randomization was determined using computer generated random numbers by a member of the lab not involved in the study. Sealed opaque envelopes (block-randomization, n = 4) were revealed to study personnel after obtaining informed written consent and completion of baseline measurements. 

### 2.3. Dietary Intervention

In the two experimental conditions, participants consumed five days of a diet consisting of 50% total energy intake (TEI) from fat, 30% of TEI from carbohydrate and 20% of TEI from protein. All food during each experimental condition was provided to participants and daily food checklists were completed to maximize compliance. However, the timing of energy intake differed between conditions: meals were consumed between 0700 and 2200 h for a 15 h controlled extended feeding (EXF) window; and between 1000 and 1800 h for an 8 h time-restricted feeding (TRF) window. Participants were instructed to consume meals at standardized times throughout both experimental conditions (EXF, within ±30 min of 0700, 1400 and 2100 h; TRF, within ± 30 min of 1000, 1300, 1700 h; Figure 1) and no other meals or snacks were provided or consumed by participants during the intervention. Daily energy distribution for both experimental conditions was aligned with reported habits of the Australian population [21], that is 25% TEI at breakfast, 30% TEI at lunch and 45% TEI at dinner, with each meal having the same macronutrient composition as the total daily composition (Table 1, Table S1). Nutrient composition of all foods was obtained from FoodWorks© Software (Version 8, Xyris, QLD, Australia). Total daily estimated energy intake was calculated using resting energy expenditure (REE) measured at baseline × an activity factor of 1.4 to provide estimated energy intake in kcal/day.

Where participants were habitual caffeine consumers (at least one caffeinated beverage/d for 7 d/wk), they were instructed to consume caffeine (no added milk or sugar/sweeteners) between 0700–1200 h (EXF) or 1000–1200 h (TRF) only during the five-day experimental periods. Participants consumed water *ad libitum* and were instructed to record daily fluid intake. On Day 5 of the experimental conditions, participants stayed in the laboratory for a continuous 24-h period (day and night) during which serial blood samples were taken (as described). During this stay, participants consumed meals according to the same feeding pattern as the previous four days. 

### 2.4. Study Protocol 

At the baseline visit, participants presented fasted (>10 h) for assessments of BM (SECA 703 scales, Hamburg, Germany), body composition via dual-energy x-ray absorptiometry (DXA; GE Lunar iDXA Pro, enCORE software Version 16) and REE (25 min duration; TrueOneRMR, Parvo Medic, Sandy, UT, USA), as previously described [22]. Participants were provided with a three-day food diary to record all food and fluid consumed (Thursday–Saturday) prior to their first experimental condition. Participants were instructed to record quantities using household measures, and to report the time of consumption to establish dietary habits at baseline. Each participant was provided with their food record from the first condition to replicate in the three days prior to the second condition. 

On Day 0 (Saturday), participants reported to the laboratory and provided the study dietitian with their three-day food records that were subsequently assessed for completeness and accuracy, with further clarification with each participant if required. A continuous glucose monitor system (CGMS; iPro2 CGM with Enlite Sensor, Medtronic, Northridge, CA, USA) was inserted into the subcutaneous tissue of the lower back and was worn continuously until the morning of Day 6. A hand-held glucometer (Accu-Chek Performa II, Roche Diagnostics Ltd., Switzerland) was provided to each participant for CGMS calibration four times/day (upon waking, pre-lunch, pre-dinner and before sleep). In order to assess physical activity patterns throughout the study periods, participants were fitted with an ActivPAL3^TM^ inclinometer (tri-axial physical activity monitor, PAL-technologies Ltd., Glasgow, Scotland) adhered to the thigh and an ActiGraph GTX3+ accelerometer (Pensacola, FL, USA; during waking hours only) worn over the right hip and fastened around the waist. All monitors were worn from Day 0 and to the morning of Day 6 of both experimental conditions. Food was provided for the first four days (Days 1–4) of an experimental condition (Table 1, Table S1) relative to each participant’s energy requirements, in clearly labelled bags for each day (Days 1–4) and meal (breakfast, lunch and dinner). Each participant was provided with a handbook to record dietary compliance (time and checklist for consumption), sleep/wake times and activity monitor removals. Participants were not provided any guidance on sleep times but were required to adhere to the dietary intake times and self-report sleep duration and quality in their handbook. 

On the day of an experimental condition (Day 5), participants in groups of 2–5 arrived at the laboratory at ~0630 h after an overnight fast where they remained until 0730 h the following day (Day 6). Meals were consumed at the times that corresponded to the allocated experimental condition (Figure 1). Specific to each mealtime, participants were permitted a 10 min walk around the university campus (~700 m) 1 h after each meal (i.e., 3 × day). Aside from these structured walk periods, participants were “free-living” in the lab where they could sit, stand or move freely throughout the day. During the 24 h laboratory visit, blood samples (4 mL EDTA (Grenier Vacuette) and 2 mL EDTA+additives (BD P800)) were obtained, from an in-dwelling venous cannula (21G; Terumo, Tokyo, Japan) inserted into an antecubital vein, hourly between 0700 and 2300 h and every 2 h between 2300 and 0700 h of Day 6 (Figure 1). Throughout the 24 h sampling period, cannulas were kept patent with regular saline (0.9% NaCl) flushes. 

At ~2230 h participants were taken to a Nursing Simulation Suite at ACU for the overnight portion of the 24-h trial period. Each participant had their own private space (i.e., bed and surrounds). Bloods taken from 2300 h were sampled whilst the participants were supine and asleep, when possible. After the ~0700 h waking blood sample, participants removed their activity monitors and CGMS before leaving the laboratory. After a 10-day washout period, the Day 0 and Day 5–6 visits were repeated for the second condition. 

### 2.5. Qualitative Interviews and Analysis

Semi-structured interviews were conducted by the same investigator (BLD) to gain insight and understanding of the attitudes of participants to both the TRF and EXF dietary conditions. The interviews were conducted face-to-face by the same investigator who recorded all responses (written record) on the morning after the second (final) condition. Table 2 outlines each interview question, including inquiry logic for generating the information. The questions within the interviews were developed in consultation with researchers and dietitians to gain insight into the attitudes and opinions, potential barriers, likelihood of adhering to a TRF dietary approach long term and influence of TRF dietary pattern on hunger levels. The number of questions was chosen to ensure interviews were timely but still explored the topic in sufficient depth.

Following each qualitative interview, written responses were read several times by the same investigator who conducted the interview to become more familiar with the answers. Following this, thematic coding of transcripts and notes was completed and organized into categories with common themes. Post-analysis verification of themes was conducted between two investigators (BLD and BER) to identify common opinions amongst participants. Quotations from original responses have been presented in the Supplementary Material to provide support for the findings.

### 2.6. Data Analysis 

Habitual dietary intake from the baseline recording periods was analyzed using FoodWorks© Software (Version 8, Xyris, QLD, Sping Hill, Queensland, Australia). All foods and beverages were analyzed, excluding vitamin and mineral supplements. Mean energy, macronutrient (carbohydrate, protein, fat), alcohol, fiber and sodium intake were obtained. The number of eating occasions over a 24-h period, and the times of eating were also recorded. Breakfast was defined as food/fluid consumed between waking and 1100 h, lunch between 1100 and 1500 h, and dinner between 1500 h and bedtime. All activity monitor analysis was performed using SAS 9.4 (SAS Institute, NC, USA), as previously described [22]. 

Total area under the curve (AUC_total_) was calculated for venous glucose, insulin, triglyceride and NEFA concentrations using the trapezoid method using GraphPad Prism (Version 7.01, GraphPad Software Inc., CA, USA). Nocturnal (sleep) AUC_total_ for glucose was calculated from periods of sleep (2300 h) to wake (0700 h) during each condition, with waking glucose from 0700 to 2300 h, inclusive. For CGMS (n = 10 due to sensor detachment for one participant), 24-h data from 0700 h on Day 5 to 0700 h on Day 6 was used to calculate mean and peak glucose and AUC_total_, for the final 24 h of each condition. Using the mealtimes, 3 h postprandial periods were calculated for venous glucose, CGMS glucose and insulin for incremental AUC (iAUC_meal_; trapezoid method with pre-meal glucose as baseline). 

### 2.7. Biochemical Analysis 

Upon collection, blood samples (EDTA) were inverted and blood glucose analyzed in whole blood at the time of collection using an YSI 2900 analyzer (YSI Life Sciences, Yellow Springs, OH, USA; coefficient of variation (CV) < 1.0%). During this time, EDTA+additive tubes were spun at 1000 *g* for 10 min at 4 °C and the resulting plasma was aliquoted and stored at −80 °C for subsequent analysis. The remaining EDTA blood sample was centrifuged at 1800 *g*, for 10 min at 4 °C and plasma was aliquoted and stored as above. From thawed plasma samples, triglyceride concentrations were measured using a Cobas Integra 4000 instrument (Roche Diagnostics Ltd., Switzerland). Insulin, non-esterified fatty acids (NEFA) and cortisol concentrations were measured using enzyme-linked immunosorbent assay (ELISA; Insulin: Alpco Ltd., Windham, New Hampshire, USA; NEFA: Wako Pure Chemical Industries, Ltd., Osaka, Japan; Cortisol: Enzo Life Sciences Inc., Farmingdale, USA) with intra-assay CV’s of 4.5%, 6.3% and 2.9%, respectively. From the thawed EDTA+additive plasma samples, connecting peptide (C-peptide), gastric inhibitory peptide (GIP), peptide tyrosine tyrosine (PYY), leptin and total glucagon-like peptide 1 (tGLP-1) concentrations were measured (excluding 0100 and 0500 h sample time points due to plate size) using a Luminex Analyzer (MAGPIX^®®^; Human Metabolic Hormone Magnetic Bead Panel, EMD Millipore, MA, USA) following manufacturer’s instructions with intra-assay CV’s of 2.2%, 2.7%, 3.4%, 4.2% and 4.2%, respectively. 

### 2.8. Statistical Analysis 

The measures reported here were taken as part of a larger study where the primary outcomes are under review, therefore no *a priori* power calculations were performed for this analysis. Statistical analysis was undertaken using SPSS (Version 25 for Windows, SPSS Inc., Chicago, IL, USA). Data from the two conditions was analyzed using Linear Mixed Models (LMM) for changes between the TRF and EXF conditions, with glucose total AUC as the primary outcome variable. All models used an identity variance-covariance structure with fixed effects (time (time-of-day; where required) and sequence effects (condition order)) and were adjusted for potential covariates explaining residual outcome variance (age and BMI). Where significant, post-hoc tests were performed using Bonferroni corrections. Due to the exploratory nature of the current analyses and the importance of identifying variables of interest for future investigations, we acknowledge there may be the potential for Type II errors of multiple comparisons with the small sample size. Significance for main effects was set at *p* < 0.05 and all data are presented as mean ± SD, with mean differences ± SD of differences between conditions when significant.

## 3. Results

### 3.1. Participant Characteristics

**Figure 2:**
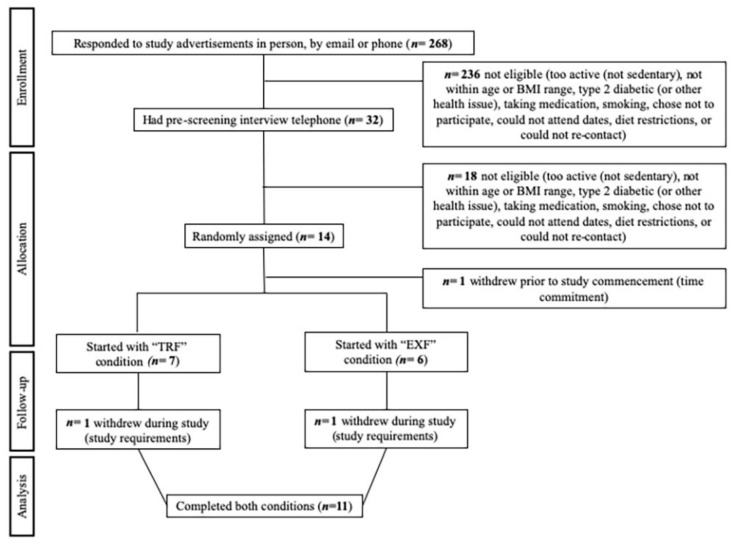
Consolidated Standards of Reporting Trials (CONSORT) flow diagram of participant recruitment.

Of the 32 individuals who were phone-screened, 14 eligible participants consented and 13 participants were randomized (Figure 2). Eleven participants (mean ± SD, age 38 ± 5 y; BMI 32.2 ± 2.0 kg/m^2^; BM 103.2 ± 9.3 kg; body fat percentage 34.0 ± 3.6%) completed both conditions with no differences between baseline variables for completers compared to non-completers (n = 3). The MEQ-SA group score was 58 ± 9, with participants indicating “definite morning” (n = 2; 70–86), “moderate morning” (n = 3; 59–69) or “intermediate” (n = 6; 42–58) types. No adverse effects were observed or reported. 

### 3.2. Dietary Compliance

All participants reported consuming the provided meals and adhered to the time guides and caffeine restrictions for the four unsupervised days (Table S1). 

### 3.3. Glucose, Insulin and Lipid Metabolism

**Figure 3:**
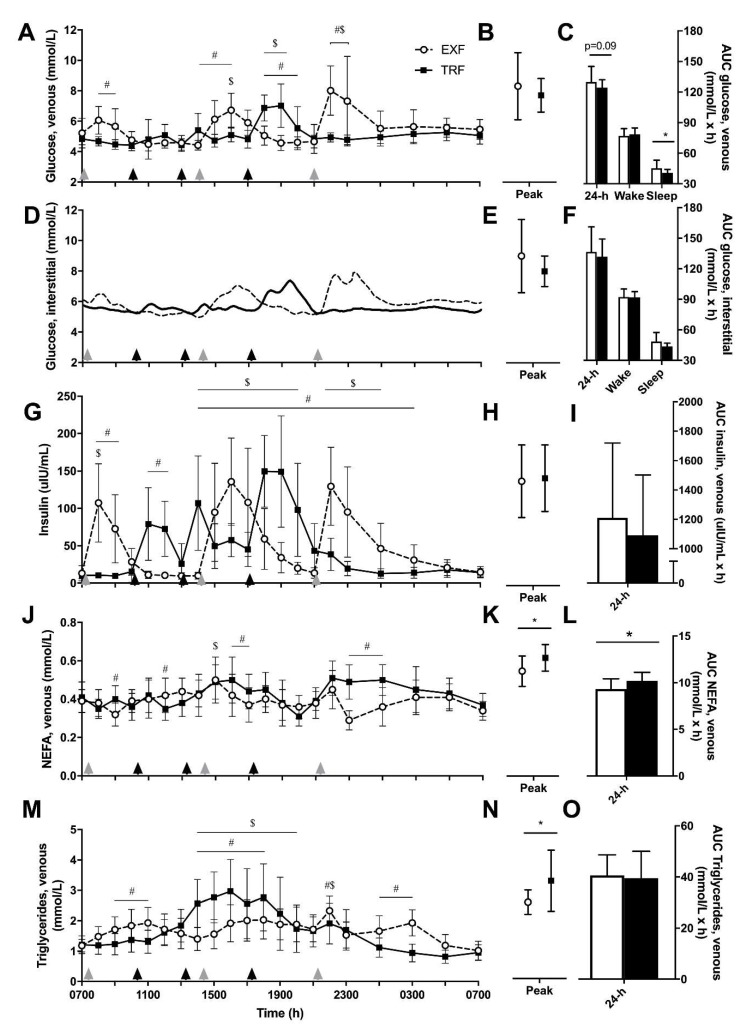
Venous glucose (**A**–**C**; n = 11), interstitial glucose (**D**–**F**) and venous insulin (**G**–**I**), non-esterified fatty acids (NEFA; **J**–**L**), triglyceride (**M**–**O**) concentrations, peak concentrations and total area under the curve (AUC) values, respectively, from participants with overweight/obesity (n = 10) throughout trial conditions of time-restricted feeding (TRF; black squares and lines) and extended feeding (EXF; white circles and dotted lines). Black triangles represent TRF mealtimes whereas grey triangles represent EXF mealtimes. Data are mean ± SD. *p* < 0.05 for * main effect of condition; # significantly different between conditions within time point; $ significantly different to baseline (0700 h; effect of time).

A main effect of condition (*p* = 0.03) as well as time and interaction effects (*p* < 0.001) were observed for venous glucose concentrations (Figure 3A), with differences between conditions at each of the meal time points revealed by post-hoc tests. No difference in daily peak glucose concentrations was detected (Figure 3B). Total 24-h area under the curve (AUC_total_) for venous glucose tended (*p* = 0.09) to be lower for TRF compared to EXF (−5.5 ± 9.0 mmol/L/h), which was largely caused by nocturnal (sleep) glucose AUC being lower in the TRF condition (−4.2 ± 5.8 mmol/L/h, *p* = 0.04), with no difference in waking glucose AUC (*p* = 0.16; Figure 3C). Due to the multiple measurement points (n = 288), the interstitial glucose × time was not subjected to statistical analyses but has been presented for comparison with venous glucose (Figure 3D). AUC_total_ from interstitial (CGMS) glucose concentrations were similar between conditions for 24-h, waking and sleep time periods, and between peak glucose concentrations (Figure 3E–F).

Venous insulin concentrations showed a time × condition interaction and a main effect of time (*p* < 0.001; Figure 3G), where insulin concentrations were different between conditions from mid afternoon (1400 h) to early morning (0300 h). AUC_total_ for venous insulin concentrations tended to be lower, but was not significantly, in the TRF condition (*p* = 0.11; −114 ± 197 mIU/mL/h; Figure 3I) with no differences in peak insulin concentrations (*p* = 0.28; Figure 3H). A time × condition interaction with main effects of condition and time were measured for NEFA concentrations (all *p* < 0.001; Figure 3J), where NEFA concentrations were elevated in the TRF condition in the early (1800–1900 h) and late (2300–0100 h) evening. Further, AUC_total_ and peak NEFA concentrations were greater in the TRF compared to EXF condition (*p* < 0.001, +0.9 ± 0.5 mmol/L/h, Figure 3L; *p* = 0.02, +0.06 ± 0.07 mmol/L, Figure 2K). A time × condition interaction and a main effect of time was detected for triglyceride concentrations (both *p* < 0.001; Figure 3M), where triglyceride concentrations were elevated from 1400 to 1900 h in both conditions and there were differential responses between conditions following mealtimes. AUC_total_ triglyceride concentrations were not different (Figure 3O), but peak triglyceride concentrations were higher in the TRF compared to EXF condition (Figure 3N).

### 3.4. Meal Responses

**Figure 4:**
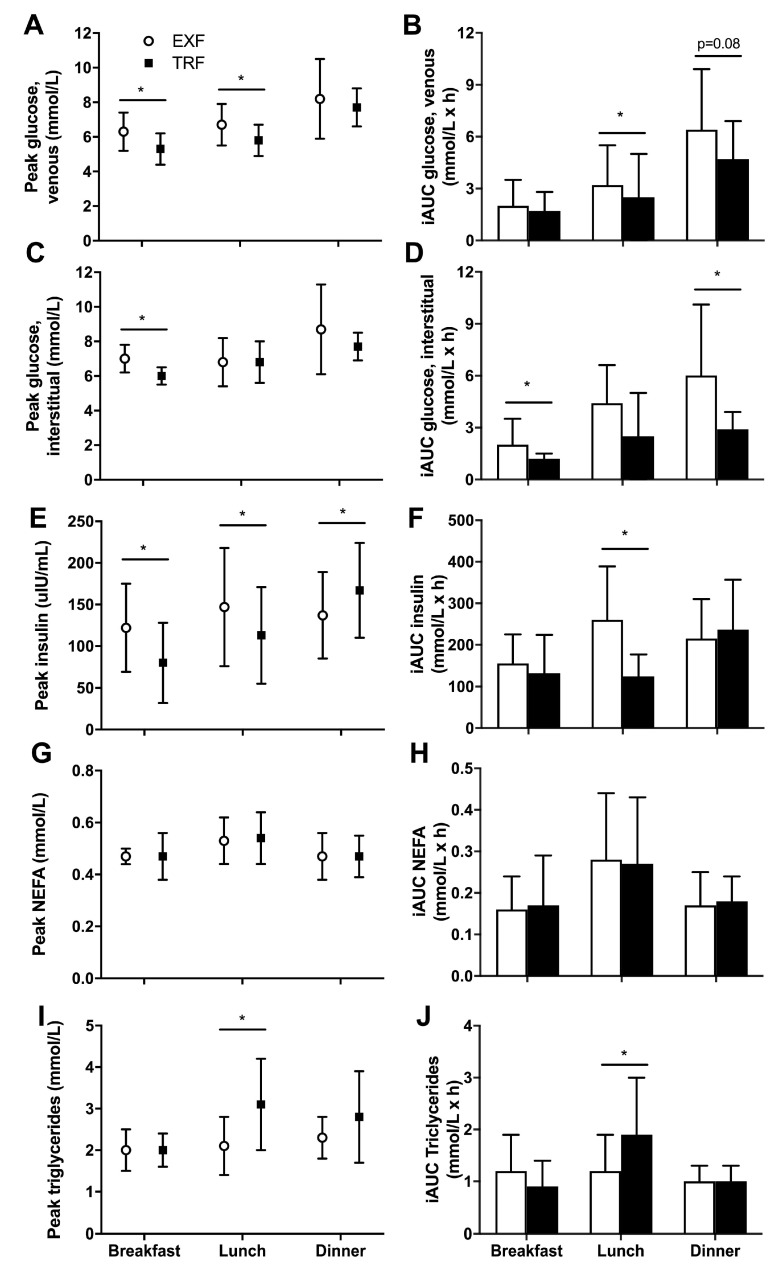
Three-hour postprandial meal peak and incremental area under the curve (iAUC) glucose (venous, **A**,**B**, and interstitial (CGMS), **C**,**D**), insulin (**E**,**F**), non-esterified fatty acids (NEFA, **G**,**H**) and triglyceride (**I**,**J**) concentrations for breakfast, lunch and dinner periods from participants with overweight/obesity (n = 10) throughout trial conditions of time-restricted feeding (TRF; black squares and bars) and extended feeding (EXF; white circles and bars). Black triangles represent TRF mealtimes whereas grey triangles represent EXF mealtimes. Data are mean ± SD. *p* < 0.05 for * main effect of condition.

Peak glucose concentration during the 3 h postprandial meal periods was lower at breakfast (*p* = 0.008; −1.0 ± 0.9 mmol/L) and lunch (*p* = 0.003; −0.9 ± 0.7 mmol/L) in the TRF condition but was similar after dinner (*p* = 0.43; Figure 4A). Incremental AUC (iAUC_meal_) for venous glucose showed the later breakfast in the TRF condition did not change iAUC_meal_ (*p* = 0.55; Figure 4B). However, the glucose iAUC_meal_ following lunch was lower in the TRF compared to EXF condition (*p* = 0.001; −2.4 ± 1.5 mmol/L/h) and tended to be lower following dinner (*p* = 0.06; −1.8 ± 2.8 mmol/L/h; Figure 4B). 

Peak interstitial glucose concentration was lower at breakfast in the TRF condition (*p* = 0.001; −1.0 ± 0.7 mmol/L), although there were no differences between conditions in peak glucose at lunch (*p* = 0.91) or dinner (*p* = 0.22; Figure 4C). The interstitial glucose iAUC_meal_ responses differed from venous glucose measurements, whereby TRF lowered iAUC_meal_ in the 3 h following breakfast (*p* = 0.04; −1.0 ± 1.2 mmol/L/h), with no differences following lunch (*p* = 0.53; Figure 4D). However, the iAUC_meal_ in the 3 h following dinner was significantly lowered in the TRF condition (*p* = 0.02; −3.2 ± 3.4 mmol/L/h; Figure 4D), and this difference was of a greater magnitude than measured with venous sampling. 

Peak insulin concentrations at breakfast (*p* = 0.05; −42 ± 61 µIU/mL) and lunch (*p* = 0.04; −35 ± 43 µIU/mL) were lower in TRF, but higher at dinner in TRF (*p* = 0.05; +33 ± 44 µIU/mL) than EXF (Figure 4E). The iAUC_meal_ insulin reflected that of venous glucose, whereby there was no difference at breakfast (*p* = 0.54) and dinner (*p* = 0.64), but the iAUC_meal_ for lunch was lower in the TRF condition, compared to EXF (*p* = 0.003; −134 ± 96 µIU/h/mL; Figure 4F). 

Peak NEFA concentrations or iAUC_meal_ were not different at any mealtime between conditions (Figure 4G-H). Peak TAG concentrations were not different at breakfast (*p* = 0.94) or dinner (*p* = 0.11), but significantly higher in TRF at lunch (*p* = 0.003, +0.9 ± 0.7 mmol/L; Figure 4I). Similarly, at lunch, the TAG iAUC_meal_ was higher in the TRF condition (P = 0.005, +0.8 ± 1.0 mmol/L/h) with no effects of condition at breakfast or dinner (Figure 4J). The trial order, included as a sequence variable when no time variable was available, was not significantly different for any AUC or iAUC measure.

### 3.5. Incretin, Appetite and Stress Hormones

**Figure 5:**
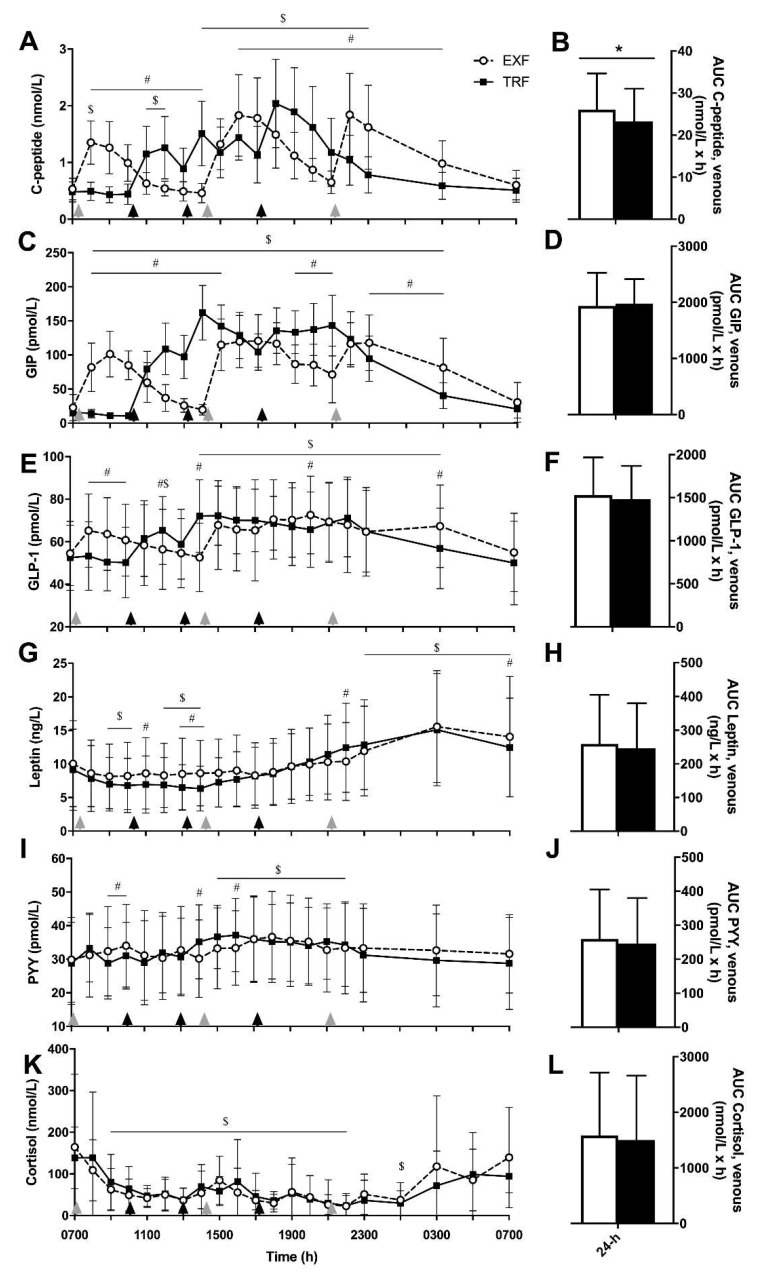
Venous c-peptide (**A**,**B**), glucose-dependent insulinotropic polypeptide (GIP; **C**,**D**), glucagon-like peptide 1 (GLP-1; **E**,**F**), leptin (**G**,**H**), peptide tyrosine tyrosine (PYY; **I**,**J**) and cortisol (**K**,**L**) concentrations over time and AUC_total_, respectively, from participants with overweight/obesity (n = 10) throughout trial conditions of time-restricted feeding (TRF; black squares and lines) and extended feeding (EXF; white circles and dotted lines). Black triangles represent TRF mealtimes whereas grey triangles represent EXF mealtimes. Data are mean ± SD. *p* < 0.05 for * main effect of condition; # significantly different between conditions within time point; $ significantly different to baseline (0700 h; effect of time).

Similar to insulin concentrations, C-peptide concentrations showed main effects of time and time × condition interactions (*p* < 0.001 for both; Figure 5A), where C-peptide concentrations were different between conditions from 1400 to 2300 h. Subsequently, AUC_total_ for C-peptide was greater in the EXF compared to TRF condition (*p* = 0.004, +2.9 ± 3.1 mmol/L/h, Figure 5B). When PYY and GLP-1 concentrations were measured, main effects of time and time × condition interactions were observed (*p* < 0.001 for all; Figure 5I and 5E, respectively), with no main effects of condition. GLP-1 concentrations were elevated above baseline from mid-afternoon (1400 h) to early morning (0300 h) but there was no effect of condition on the AUC_total_ (Figure 5F). PYY concentrations were elevated above baseline from 1500 to 2200 h, with no differences between PYY AUC_total_ between conditions (Figure 5J). 

For GIP and leptin, main effects of condition, (*p* < 0.001 for both), time (*p* < 0.001 for both) and time × condition interaction effects were measured (*p* = 0.002 and *p* < 0.001; Figure 5C and 5G, respectively). Although GIP and leptin concentrations varied over time between conditions, there was no difference between AUC_total_ between conditions for either GIP or leptin (Figure 5D and 5H, respectively). For cortisol, only a main effect of time was evident (*p* < 0.001; Figure 5F), where cortisol concentrations were lower than baseline (0700 h) from 0900 to 2200 h.

### 3.6. Subjective Appetite and Fatigue Ratings

**Figure 6:**
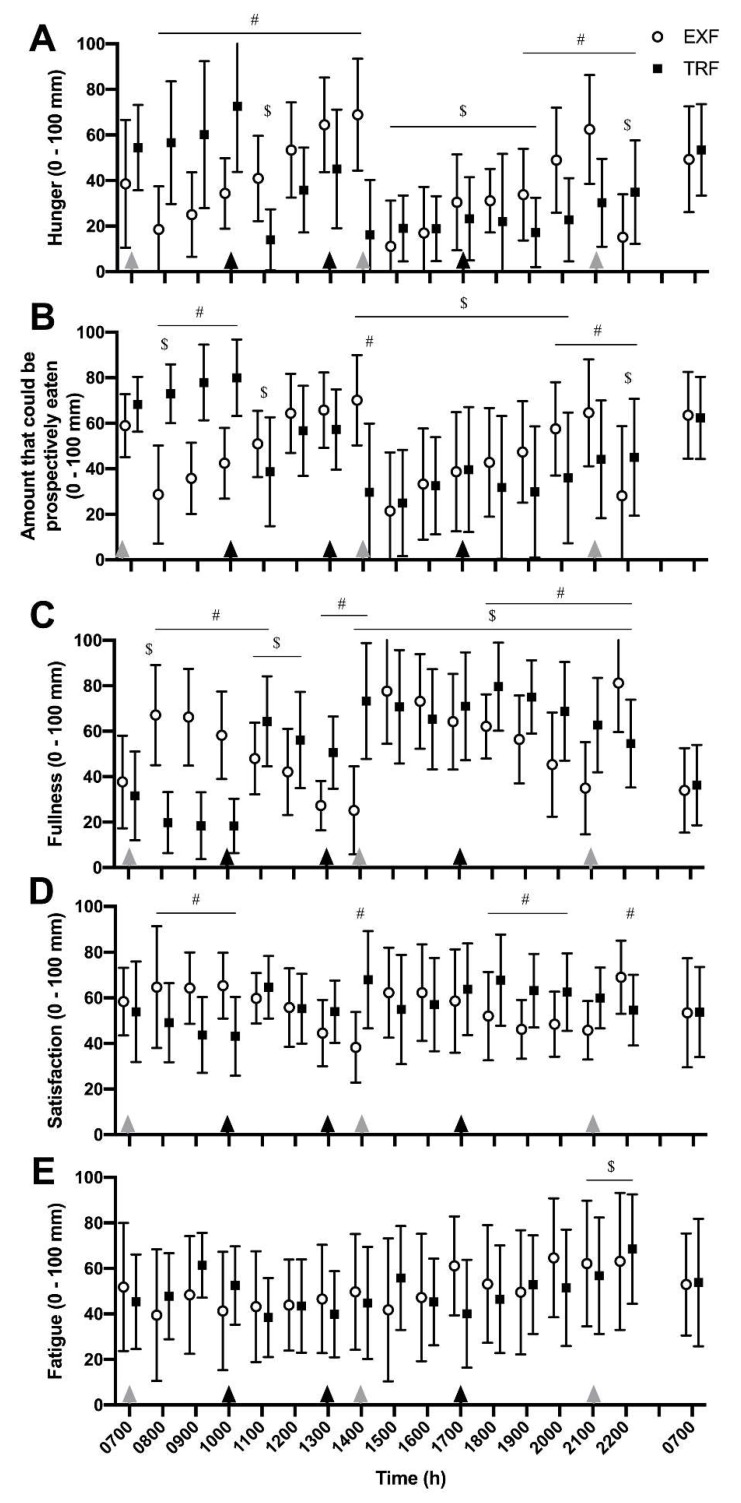
Subjective hunger (**A**), appetite (**B**), fullness (**C**), satiety (**D**) and fatigue (**E**) responses to visual analogue scale questions (0–100 mm) from participants with overweight/obesity (n = 11) throughout trial conditions of time-restricted feeding (TRF; black bars) and extended feeding (EXF; white bars). Black triangles represent TRF mealtimes whereas grey triangles represent EXF mealtimes. Data are mean ± SD. *p* < 0.05 for # significantly different between conditions within time point; $ significantly different to baseline (0700 h; effect of time).

There was a significant effect of time and a time × interaction effect (both *p* < 0.001) were observed for subjective hunger ratings, where a lower rating indicates less hunger (Figure 6A). Hunger was suppressed in both conditions from 1500 to 1900 h, but only remained suppressed in the TRF condition at 2000 and 2100 h. The delayed breakfast consumption also varied morning hunger responses (0800–1400 h) between conditions. As a measure of satiation, the amount that could be prospectively eaten was altered across time (*p* < 0.001) and a condition × time interaction was measured (*p* < 0.001; Figure 6B), where a similar suppression of amount that could be prospectively eaten was observed from late afternoon (1500 h) onwards, remained suppressed in the TRF condition only at 2000 and 2100 h, and increased morning satiation in the EXF group until 1100 h (i.e., after TRF had also had breakfast). 

Self-reported fullness was also altered across time (*p* < 0.001) with a time × condition interaction observed (*p* < 0.001; Figure 6C), with elevated fulness ratings above baseline from 1400–2300 h in both conditions and differential responses between conditions in the morning (0800–1200 h) and evening (1800–2200 h) were observed due to the different lunch times (1300 h, TRF; 1400 h, EXF). For subjective ratings of satisfaction, an interaction effect was measured (*p* < 0.001; Figure 6D), where participants reported reduced satisfaction between 0800 and 1000 h in the TRF condition compared to EXF and greater satisfaction between 1800–2000 h in TRF compared to EXF. For subjective ratings of fatigue, only a main effect of time was detected (*p* < 0.001; Figure 6E), with increased ratings of fatigue in both conditions from 2100–2200 h.

### 3.7. Qualitative Questionnaire Responses

A summary of the themes identified from participant interviews are outlined in Table 3, with supporting quotations provided in Supplementary Material. Overall, positive attitudes were expressed towards a TRF dietary pattern. Whilst four participants commented it took a couple of days to adjust to the TRF dietary pattern, the remainder felt TRF was both manageable and practical. Participants reported that they felt that adopting TRF in their normal routine would reduce non-hungry snacking and “mindless eating” in the evening. Additionally, several participants reported that they thought a TRF pattern could help add structure to their current eating patterns. Finally, participants reported enhanced feelings of well-being following the TRF diet intervention.

Based on their experience during the two dietary conditions, participants outlined three main barriers that could be problematic when attempting to adopt a TRF pattern (Table 3). All participants (n = 11) mentioned that their work schedules would make TRF adherence challenging. Participants reported their days at work are busy and sometimes they do not consume food at all during a workday. The specific timing of a TRF eating pattern (as used in this intervention) would mean a major portion of total energy intake would be eaten in the workplace, which for some individuals was not possible. Social life was identified as a barrier for majority of participants (n = 8) to the possible likelihood of sustaining a TRF pattern. Specifically, participants commented that they would find eating out with friends and family challenging if the hours to consume meals and drinks was limited to just 8 h/day. For those participants with a family (n = 5), a common theme was that family needs come first in their household, and therefore implementing a TRF pattern, particularly an early dinner, would be unpractical and extremely difficult.

The participants outlined several positive factors of a TRF feeding pattern. Specifically, participants felt that the TRF pattern improved their daily eating structure, especially in relation to work and other daily habits. Over half of participants (n = 6) commented that a TRF eating pattern reduced discretionary snacking in the evening and found it appealing that TRF could be such a simple method to reduce their food intake. Participants self-scored their ability of adhering to a TRF dietary pattern as 6.7 (range: 3–10; where a score of 1 being not at all possible and 10 being most definitely possible). Participants who gave a low rating of the likelihood of possible adherence to TRF attributed this to barriers such as social life and work schedules. Conversely, participants with high ratings of possible adherence to TRF mentioned that a TRF eating pattern helped with their daily planning and structure, reduced snacking in the evening, the potential for weight loss and improved feelings of general well-being. The participants in the current study were provided adequate food to meet their daily energy demands (both dietary conditions were isoenergetic). Accordingly, hunger was not a major barrier towards the adoption of a TRF eating pattern under the conditions of this study. 

### 3.8. Physical Activity and Habitual Dietary Intake

There were no differences between conditions for time spent in sedentary, light or moderate-vigorous physical activity, or for the proportion of waking time spent sitting, standing and stepping (Table S2). Despite instructions and providing previous food records to participants, food records analyzed from the 3-day periods prior to each 5-day dietary intervention showed a lack of replication, with greater total energy, percentage contribution of carbohydrate and absolute amounts of protein, fat and saturated fat (*p* < 0.05) in the 3-days before EXF, compared to TRF (Table S3).

## 4. Discussion

This is the first human intervention to investigate the effects of a modified TRF protocol (i.e., delaying the timing of breakfast) as a practical strategy to prolong the duration of fasting and improve markers of glycemic control in individuals with overweight/obesity. The current study is also the first to report the responses and attitudes of males with overweight/obesity towards this novel TRF strategy, including potential benefits and barriers individuals face when adopting a time-restricted eating pattern. Awareness of these barriers may help in the development of practical solutions to overcome and improve successful outcomes (i.e., compliance, weight loss). Overall, participants expressed a positive attitude towards a TRF dietary pattern along with improvements in feelings of well-being and reduced snacking in the evening.

The provision of meals to our participants consisting of the same macronutrient composition and energy throughout the day in both conditions provides “proof of concept” that a TRF strategy in which the first energy intake of the day is delayed could provide a potential adjunct therapy for improving postprandial and nocturnal glycemic control and reducing subjective appetite ratings. Our finding of a tendency for improved 24-h glycemic control was largely due to a reduced nocturnal glucose area under the curve as no difference in overall regulation of glucose during waking hours was observed. Reduced nocturnal glucose aligns with a recent 4-day intervention of eTRF (0800–1500 h eating window) in individuals with overweight and obesity in which 24-h glucose area under the curve was lower compared to a control, extended eating condition (0800–2000 h) [12], an effect largely attributable to reductions in nocturnal glucose concentrations. As greater peaks and sustained rises (i.e., postprandially and nocturnally) in glucose contribute to endothelial dysfunction and cardiovascular disease in individuals with T2DM [23], strategies such as TRF may be important for reducing the development of these complications. Our intervention not only reduced nocturnal glucose concentrations, but also lowered the postprandial glycemic response to the same breakfast meal. Accordingly, the timing of our TRF intervention (delaying breakfast in combination with an earlier dinner) serves as a practical option for integrating TRF to improve glycemic control. Future studies where TRF is investigated in patients with T2DM is of importance in light of these findings, as oral medications do not reduce the morning-related rises in glucose concentrations [24].

TRF resulted in favorable changes to insulin profiles throughout the day, with reduced peak postprandial insulin concentrations at breakfast and lunch and lowered insulin levels after lunch. As insulin sensitivity declines in the late afternoon/early evening [25,26], a TRF pattern in which the majority of daily energy is consumed earlier in the day prior to this period of reduced insulin sensitivity is a desirable goal. C-peptide is released from pancreatic *ß*-cells in equimolar amounts to insulin, but while insulin first passes through and can be metabolized by the liver, thus reducing circulating concentrations, C-peptide provides a surrogate measure of *ß*-cell function [27]. The reduced AUC_total_ for C-peptide from the TRF protocol indicates improved *ß*-cell function compared to eating in the EXF pattern. Indeed, a recent report demonstrated improved *ß*-cell function (as measured by the insulinogenic index) when meals were consumed in an eTRF (0800–1500 h) pattern for 5 weeks in men with prediabetes [13]. While peak insulin concentrations in the current investigation were significantly higher when the dinner meal was consumed early (1700 h) versus late (2100 h), there were no differences in peak glucose concentration and incremental AUC glucose was lowered when dinner was consumed earlier. Collectively, our data suggest an increased sensitivity to glucose with a TRF protocol in which dinner is consumed by 1800 h. A second meal effect [28] was observed in the TRF condition, where the lunch meal (consumed 3 h after commencing breakfast) elicited lower venous glucose and insulin concentrations. In contrast, the second meal effect was less pronounced in the extended feeding condition, due, in part, to the longer time period between meals and the reduced time of overnight fasting (9 vs. 16 h/day). However, one week of eTRF (0800–1700 h) improved fasting glucose concentrations in men at risk of T2DM compared to delayed TRF (1200–2000 h) [17]. Therefore, the glycemic status of the participant cohort strongly influences any improvements in fasting glucose concentrations. As such, short-term delayed-breakfast TRF seems to impart beneficial effects on glycemic control which is likely dependent on both the length of the overnight fast and the time of day of the eating window.

Whilst our interpretation of appetite and incretin hormones and subjective appetite ratings are exploratory, it is clear that changing the timing of eating alters subjective ratings of appetite and related hormone measurements. Participants had lower ratings of hunger and prospective food consumption from the lunch period onwards in the TRF condition, compared with a rise prior to the late evening meal in the extended feeding condition. Taken collectively, these observations suggest TRF may be a useful strategy for reducing afternoon/evening appetite. The work of Sutton and colleagues [13] support reduced subjective ratings of appetite. However, their results are difficult to place in context as the time of questioning of participants was after dinner in their eTRF condition and before eating dinner in the control condition. Therefore the appetite ‘response’ cannot be directly associated with the eTRF protocol per se. However, hunger at bedtime has also been reported to be reduced after 16 weeks of self-selected 10 h TRF [10]. How such changes to hunger and appetite then translate to potential long-term changes to eating behaviors warrants further investigation. 

There are several aspects of our study design which may inform the optimal timing of a TRF intervention to improve daily glycemic control. First, delaying breakfast by 2–3 h avoids the early morning rises in glucose and insulin concentrations and results in a lower AUC response compared to when an identical meal is consumed earlier in the day. Concomitant with the increase in circulating glucose and insulin upon waking, peaks in cortisol occur around the time of breakfast (~0800 h) [26] and were not altered by the changes in eating times over 5 days. High levels of circulating cortisol stimulate gluconeogenesis and glycogenolysis resulting in increased blood glucose concentrations [29]. Therefore, a delayed breakfast avoids peak cortisol levels which may contribute to higher peak glucose concentrations when breakfast is consumed earlier. Second, consuming a late evening meal (after 2000 h) has been shown to increase the postprandial blood glucose response to carbohydrate at breakfast [30], linked with a slower motility of the gastrointestinal tract [31]. Therefore, consuming an earlier dinner meal induces a different metabolic response to the same meal due to typical oscillations of insulin and glucose in response to the time of eating, but also has physiological effects stemming through to the next day’s breakfast meal that, over time, may be linked to the development of metabolic disease.

TRF limits food intake in the later evening (i.e., after 1800 h), reducing the quantity of “time-of-day dependent foods” such as alcohol and sweets [10]. The evening is often a challenging time for individuals when trying to adhere to dietary interventions or follow dietary advice. As individuals with obesity tend to consume more energy in the evening compared to lean individuals [32], TRF may result in an improved “health profile” simply because it induces a voluntary reduction in energy intake, as observed in other interventions. As such, implementing a TRF dietary pattern may be a simple and effective strategy to overcome post-dinner snacking, reduce total daily energy intake and improve feelings of well-being, factors likely to increase dietary adherence and subsequently long-term health outcomes.

Higher fat, lower carbohydrate containing diets have been associated with improved metabolic health profiles in humans [33] and thus are often recommended for improving the glycemic control of individuals with T2DM [34]. As such, we chose to investigate a moderately high-fat (50% total energy intake), moderate carbohydrate diet (30% total energy intake), in this investigation. The elevated triglyceride and NEFA concentrations in the TRF condition were a predictable outcome, due partially to the high dietary intake of fat. Unlike glucose and insulin concentrations, NEFA and triglycerides concentrations peaked higher in the TRF condition, possibly due to a whole-body lipid level imbalance with meals consumed in close (time) proximity. Similarly, an elevated level of fasting lipids was measured in a well-controlled 8-week study of eTRF in men with prediabetes [13]. 

Modification of the time of eating may induce changes in patterns of daily physical activity. In the current investigation, changing the ‘window’ in which participants consumed meals in Days 1–4 had little effect on daily physical activity. The analysis of participant’s 3-day food records prior to each experimental period raises concerns regarding the validity and reliability of compliance to ‘habitual control’ periods. Strict dietary control via provision of meals 4 days prior to the 24 h measurement day in our intervention ensured that any potential effects of habitual dietary differences would have been washed-out prior to outcome measures being taken. The issue of compliance highlights that if individuals are unable to follow their own habitual meals, then adherence to dietary restrictions (i.e., reductions in energy intake or alterations in macronutrient consumption) are highly unlikely. In studies investigating diets targeting weight loss, adherence is strongly correlated with greater weight loss [35]. As such, TRF seems to offer a more practical solution to encourage dietary adherence with minimal instruction or education and is supported by previously reported positive effects of TRF on weight loss [15,36]. 

Despite an overall positive attitude towards TRF eating pattern, several barriers to adopting the TRF eating pattern were highlighted. Participants reported busy work schedules, family meal schedules and restrictions placed upon eating at social occasions as the main barriers to TRF. In the current study, the evening meal in the TRF condition was consumed between 1700–1730 h. The early dinnertime is likely to contribute to difficulties in eating early leading to disrupted family schedules, thereby creating a practical barrier to long-term compliance. By slightly extending the TRF period to finish later in the evening (i.e., by 1900 h), this may help overcome these barriers yet still help reduce late evening snacking behaviors leading to improved glycemic control. The TRF condition also limited social activities in the evening due to the early evening meal. A potential solution to help offset some of the social barriers to eating could be to recommend that TRF be undertaken for five of the seven days of the week (i.e., during the working week but not on weekends). In rodents, adherence to a TRF eating pattern only on weekdays still had a positive effect on weight loss [9,37] and, whilst similar studies need to take place in humans, reducing TRF to five of the seven days of the week may improve adherence to a TRF dietary pattern.

Adherence to dietary recommendations/advice is crucial for long-term successful outcomes such as weight loss or changes in body composition [5,6]. Strategies to increase dietary adherence are just as important as focusing on nutrient composition of the dietary pattern itself in promoting sustained weight loss [6]. In a study comparing dietary adherence and weight loss success between three popular weight loss diets with specific macronutrient goals, it was reported that an adherence score was significantly correlated with 12-month weight change, regardless of the assigned diet [6]. Therefore, feasibility should be the initial focus of any diet intervention. As such, TRF seems to be a practical solution where the barriers identified in this study can be addressed with the aim to improve adherence and consequently successful health outcomes.

A major strength of the current investigation is the dietary control over the 24 h measurement period and the individualized provision of all meals to participants. Combining continuous glucose monitoring with 24-h venous sampling, we have been able to provide a daily snapshot of glycemic responses through postprandial periods of all meals as well as overnight. However, we acknowledge several limitations of our study that preclude us from making generalized recommendations. These include a small sample size, a male only cohort, a moderately high-fat diet, a lack of pre-study investigation of insulin sensitivity and a purposefully structured extended feeding group with no snacking or ad libitum intake. In this regard, it is likely that most individuals who eat over a longer time period (i.e., an unrestricted feeding pattern) would snack or eat smaller meals across a day, which would be likely to change the glycemic and hormonal response. We also inadvertently reduced and controlled physical activity during the 24 h laboratory stay, as an unrestricted activity pattern and/or greater amounts of physical activity is likely to alter glycemic responses [38]. 

## 5. Conclusions

In conclusion, we demonstrate the potential for modified TRF protocol in which the first eating occasion of the day was delayed by 3 h to 1000 h, to improve nocturnal and postprandial glycemic control. Such a protocol was well accepted as a dietary strategy in sedentary males with overweight/obesity. To further elucidate the beneficial effects of a TRF pattern where breakfast is delayed, glycemic control needs to be measured in varied cohorts of larger sample sizes over longer time periods, as well as compared to multiple eating occasion in individuals with obesity. The TRF protocol induced lower peak glucose and insulin responses during postprandial breakfast and lunch periods and holds promise for the improvement of daily glycemic control in those with prediabetes or T2DM. The lack of adherence to, and success of, current diet modification recommendations at the population level for reducing the ‘diabesity’ epidemic warrant focus on alternative dietary strategies such as TRF. We hope that this investigation allows for future research to further support the applicability of TRF as an alternative, potential long-term dietary strategy in the management of obesity and glycemic control.

## Figures and Tables

**Table 1:** Dietary composition of experimental diets and breakdown of meals provided to participants for 5 days in both conditions.

	Total Day	Breakfast	Lunch	Dinner
Energy (kJ)	12218 ± 1224	2989 ± 299	3715 ± 372	5505 ± 552
CHO (g)	242.9 ± 24.3	58.2 ± 5.8	71.3 ± 7.1	112.0 ± 11.2
Sugars (g)	87.3 ± 8.7	35.8 ± 3.6	9.7 ± 1.0	42.0 ± 4.2
CHO (% TEI)	30 ± 0	30 ± 0	30 ± 0	30 ± 0
Protein (g)	136.7 ± 13.7	32.0 ± 3.2	46.5 ± 4.7	56.7 ± 5.7
Protein (% TEI)	20 ± 0	20 ± 0	20 ± 0	20 ± 0
Fat (g)	162.9 ± 16.3	73.9 ± 6.2	74.1 ± 6.3	75.3 ± 6.4
Fat (% TEI)	50 ± 0	50 ± 0	50 ± 0	50 ± 0
Saturated fat (g)	77.1 ± 7.7	10.9 ± 1.1	23.6 ± 2.4	42.8 ± 4.3
Polyunsaturated fat (g)	11.6 ± 1.2	6.0 ± 0.6	2.5 ± 0.2	2.5 ± 0.2
Monounsaturated fat (g)	52.4 ± 5.2	22.7 ± 2.3	8.6 ± 0.9	20.8 ± 2.1
Fibre (g)	42.2 ± 4.2	5.1 ± 0.5	15.1 ± 1.5	22.0 ± 2.2

CHO, carbohydrate; TEI, total energy intake. Data are mean ± SD.

**Table 2:** Semi-structured interview questions and inquiry logic.

Inquiry Logic	Questions
To determine broad opinions and attitudes towards a time-restricted dietary pattern	Based on your experience throughout the study period of consuming food between 10 a.m. and 6 p.m., what are your general thoughts and comments about such a time-restricted dietary pattern? Do you have any comments, thoughts or opinions you would like to share regarding the dietary approach of consuming food in an 8-h window each day?
Explore and identify potential barriers of time-restricted feeding	What are the potential barriers to adhering to a time-restricted feeding pattern?
Explore factors that may be appealing regarding a time-restricted feeding pattern	What is appealing about such a dietary pattern?
Likelihood or possibility of adhering to such a dietary pattern	Considering your home/work/life setting, on a scale of 1–10 (1 being not possible and 10 most definitely possible), how would you rate your ability to adhere to a time-restricted approach to eating? And why did you provide this ranking?
To establish if a time-restricted feeding pattern had a strong influence on hunger levels which may influence adherence	How did you find the eating patterns influenced your hunger levels? Were there particular times of the day you were full or hungry?

**Table 3:** Summary of themes from qualitative participant interviews.

Area of Inquiry	Summary of Themes
Broad opinions and attitudes towards a time-restricted dietary pattern	Positive attitudes towards TRF including feelings of increased energy, improved feelings of well-being and reduced snacking in the evening Had difficulty adjusting to late breakfast/early dinner initially but no issues by the last day Liked the structure the TRF provided
Potential barriers of time-restricted feeding	Main barriers identified include: Work schedules Social life Family life schedules.
Appealing elements of a time-restricted feeding pattern	Structure and routine encourage good habits Reduces snacking at all hours Simple method to reduce food intake
Likelihood or possibility of adhering to a TRF dietary pattern (on a scale of 1–10 (1 being not possible and 10 most definitely possible), how would you rate your ability to adhere to a time-restricted approach to eating?)	Mean score of 6.7/10 (range: 3–10) Low ratings due to: Social life and work barriers. High ratings due to: Preference for structure and improved feelings of well-being when on TRF pattern.
Influence of TRF on hunger	Hunger not a major barrier towards TRF if adequate food consumed within the time window of intake.

## Supplementary Materials

The following are available online at https://www.mdpi.com/2072-6643/12/2/505/s1, Table S1: Example food intakes and timing between conditions; Table S2: Activity monitor analyses over the at home (Days 1-4) period in sedentary individuals with overweight/obesity in response to an unrestricted feeding (URF) and a time-restricted feeding (TRF) dietary pattern; Table S3: Habitual dietary intake analysis prior to time-restricted feeding (TRF) and unrestricted feeding (URF) trials from three day food diary analysis; Figure S1: Total area under the curve glucose from venous (A) and interstitial CGM (B) measurements for each individual participant.
