# Supplementary material for: A Delayed Morning and Earlier Evening Time-Restricted Feeding Protocol for Improving Glycemic Control and Dietary Adherence in Men with Overweight/Obesity: A Randomized Controlled Trial
Source: Nutrients. 2020 Feb 17;12(2):505. doi: 10.3390/nu12020505 (PMC7071240; doi:10.3390/nu12020505)
Supplement: Supplementary file 1 [file nutrients-12-00505-s001.zip › RESTRICT Glycaemia and quals Supplementary materials R1.docx]

**Supporting Information**

**Supplementary Methods**

*Dietary methodology*

**Table S1. Example food intakes and timing between conditions**

|  |  |  | **Condition** | |
| --- | --- | --- | --- | --- |
| **Meal** |  | **Day** | **Time-restricted feeding** | **Control** |
| **Breakfast** | **Meal time, h:min**  **(time spent eating, min)** | **Days 1 – 4** | 10:04 ± 0:06  (18 ± 15) | 07:08 ± 0:09  (18 ± 13) |
|  |  | **Day 5** | 10:12 ± 0:08  (11 ± 3) | 07:17 ± 0:08  (14 ± 8) |
|  | **Foods provided*** | **Days 1 – 5** | Almond butter pancakes (3 pancakes)  Peanut butter (32 g)  Chobani yoghurt Coconut flavoured (181 g) | |
| **Lunch** | **Meal time, h:min**  **(time spent eating, min)** | **Days 1 – 4** | 13:07 ± 0:05  (25 ± 24) | 14:05 ± 0:07  (17 ± 9) |
|  |  | **Day 5** | 13:14 ± 0:09  (21 ± 9) | 14:14 ± 0:10  (18 ± 8) |
|  | **Foods provided*** | **Days 1, 3 & 5** | White bread (160 g)  English ham 97% fat free (44 g)  Reduced fat tasty cheese (36 g)  Cheese & chive muffin (3 muffins) | |
|  |  | **Days 2 & 4** | Focaccia bread (123 g)  Shredded chicken breast (29 g)  Reduced fat tasty cheese (29 g)  Chocolate brownie (102 g) | |
| **Dinner** | **Meal time, h:min**  **(time spent eating, min)** | **Days 1 – 4** | 17:09 ± 0:09  (30 ± 35) | 21:03 ± 0:04  (23 ± 9) |
|  |  | **Day 5** | 17:15 ± 0:10  (21 ± 9) | 21:12 ± 0:08  (18 ± 5) |
|  | **Foods provided*** | **Days 1 & 3** | Beef sausages (4 sausages)  White bread (145 g)  Tomato sauce (29 g)  Frozen carrot, corn & broccoli mix (218 g)  Coconut lemon fat bombs (3 bombs)  Jelly snakes (29 g) | |
|  |  | **Days 2,4 & 5** | Chicken picata (218 g)  White rice (130 g)  Cauliflower cheese bake (116 g)  Jelly snakes (102 g) | |

* based on average energy intake band of 2,900 kcal.

**Supplementary Results**

**Table S2.** **Activity monitor analyses over the at home (Days 1-4) period in sedentary individuals with overweight/obesity in response to an unrestricted feeding (URF) and a time-restricted feeding (TRF) dietary pattern.**

| **Measure** | **URF** | **TRF** | **Difference (95%CI)** | ***P*** |
| --- | --- | --- | --- | --- |
| Sitting (%)^a^ | 71 ± 9 | 69 ± 8 | -1.4 (-9.2 – 6.3) | 0.649 |
| Standing (%)^a^ | 20 ± 8 | 22 ± 7 | 1.2 (-6.1 – 8.6) | 0.632 |
| Stepping (%)^a^ | 9 ± 3 | 9 ± 2 | 0.2 (-1.5 – 1.9) | 0.995 |
| Sitting 30 min blocks (%)^a^ | 310 ± 154 | 240 ± 85 | -120 (-241 – 1) | 0.266 |
| Steps (n)^a^ | 3089 ± 1252 | 2984 ± 1121 | -675 (-1417 – 66) | 0.024 |
| Sedentary (%)^b^ | 67 ± 11 | 65 ± 10 | -1.7 (-8.4 – 5.1) | 0.580 |
| Light PA (%)^b^ | 29 ± 10 | 31 ± 9 | 1.9 (-4.7 – 8.4) | 0.581 |
| MVPA (%)^b^ | 4 ± 3 | 4 ± 2 | -0.2 (-1.4 – 1.0) | 0.899 |

^a^From activPAL and ^b^ActiGraph activity monitors. MVPA, moderate-vigorous physical activity; PA, physical activity. Data are mean ± SD.


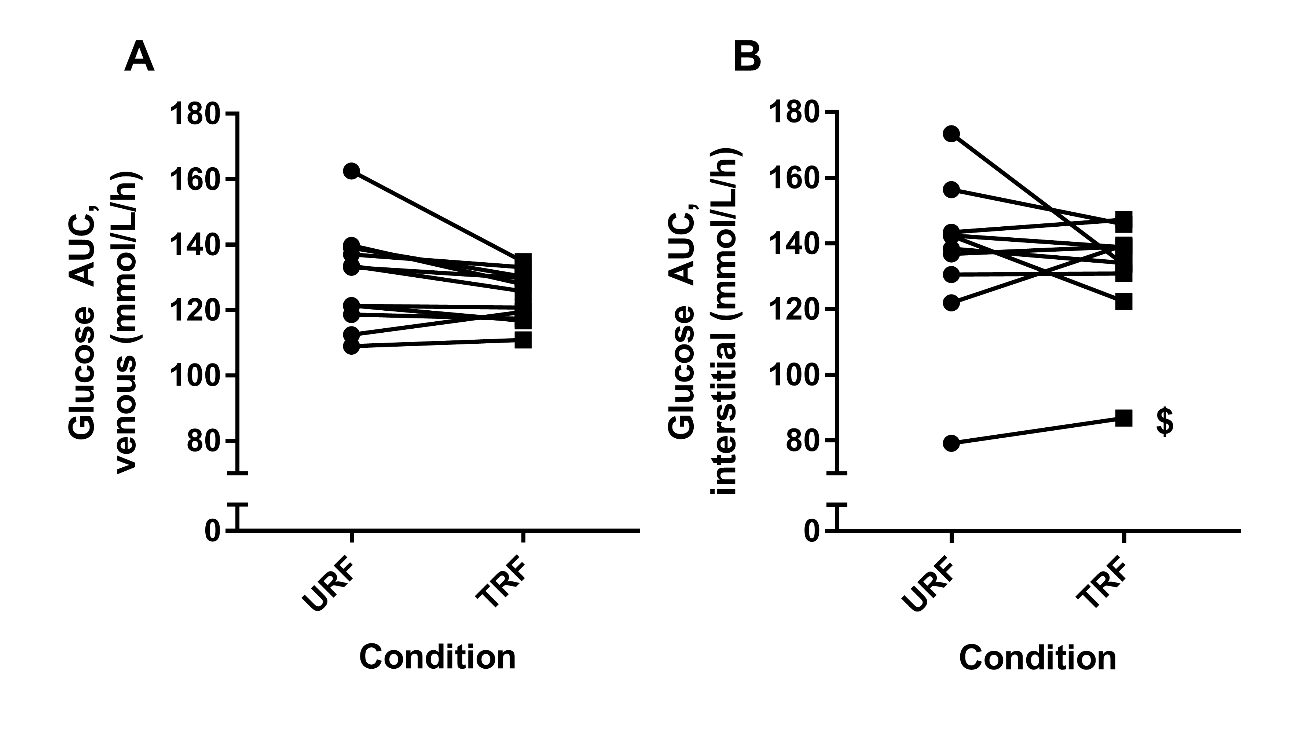


**Figure S1. Total area under the curve glucose from venous (A) and interstitial CGM (B) measurements for each individual participant.** Key: $ = CGM monitor error so only data until 0000 h in both conditions was included.

When the total AUC data is presented to show individual variability (**Figure S1**), it is clear that one individual is driving the change between conditions. Removal of this individual from the AUC_total_ analysis changes the significance from P=0.088 to P=0.092.

Overall, when comparing the dietary intake during habitual periods, participants consumed more total energy, percentage contribution of carbohydrate, and absolute amounts of protein, fat and saturated fat (P<0.05; **Table S3**) in the 3-days before EXF, compared to TRF. Consequently, the proportion of energy from carbohydrate was lower in the habitual period before EXF, compared to TRF (**Table S3**). Habitual meal analysis revealed small breakfast and lunch intakes (18 ± 11% and 28 ± 7% TEI, respectively) compared to dinner (54 ± 13% TEI). Participants’ breakfast meal composition and energy intake did not differ between habitual periods. However, the lunch energy intake was lower preceding EXF compared to TRF (-5.0, P=0.001). Accordingly, dinner energy intake preceding EXF was greater (+8.2%, P=0.006), as was the percentage contribution of carbohydrate, and absolute amounts of protein, fat, saturated fat and alcohol (P<0.05; **Table S3**).

**Table S3. Habitual dietary intake analysis prior to time-restricted feeding (TRF) and unrestricted feeding (URF) trials from three day food diary analysis.**

|  | **Prior to TRF** | | | | **Prior to URF** | | | |
| --- | --- | --- | --- | --- | --- | --- | --- | --- |
|  | **Total** | **Breakfast** | **Lunch** | **Dinner** | **Total** | **Breakfast** | **Lunch** | **Dinner** |
| Energy (kJ) | 10721 ± 1801 | 2023 ± 1215 | 3344 ± 828 | 5489 ± 2032 | 12870 ± 1558* | 2046 ± 1514 | 3148 ± 805 | 7403 ± 1621* |
| Energy  (% TEI) | -- | 19 ± 12 | 31 ± 7 | 50 ± 14 | -- | 17 ± 11 | 26 ± 7* | 58 ± 11* |
| CHO (g) | 275.6 ± 39.3 | 59.1 ± 37.5 | 106.2 ± 56.2 | 128.2 ± 39.4 | 292.1 ± 60.1 | 55.0 ± 43.3 | 82.4 ± 25.1 | 148.4 ± 47.4 |
| Sugars (g) | 97.1 ± 34.8 | 25.2 ± 16.4 | 29.6 ± 19.4 | 38.5 ± 25.9 | 101.2 ± 38.2 | 22.8 ± 16.3 | 23.2 ± 18.1 | 49.1 ± 29.1 |
| CHO  (% TEI) | 44 ± 8 | 52 ± 17 | 43 ± 17 | 42 ± 9 | 39 ± 6* | 48 ± 16 | 40 ± 12 | 36 ± 10* |
| Protein (g) | 110.7 ± 24.7 | 19.4 ± 11.7 | 30.9 ± 7.1 | 49.6 ± 13.9 | 137.0 ± 22.1* | 20.2 ± 14.7 | 34.1 ± 11.8 | 81.9 ± 19.9* |
| Protein  (% TEI) | 18 ± 3 | 16 ± 6 | 18 ± 7 | 17 ± 4 | 18 ± 3 | 16 ± 7 | 16 ± 8 | 18 ± 4 |
| Fat (g) | 105.4 ± 34.1 | 17.4 ± 13.2 | 30.6 ± 13.5 | 58.0 ± 34.7 | 130.6 ± 25.3* | 19.5 ± 17.8 | 30.8 ± 9.0 | 81.6 ± 26.8* |
| Fat  (% TEI) | 35 ± 7 | 26 ± 14 | 29 ± 14 | 37 ± 9 | 38 ± 5 | 27 ± 15 | 33 ± 8 | 38 ± 9 |
| Saturated fat (g) | 36.0 ± 12.5 | 7.3 ± 5.7 | 10.2 ± 5.1 | 18.7 ± 10.0 | 47.4 ± 13.4* | 7.6 ± 6.7 | 10.7 ± 4.3 | 29.6 ± 10.6* |
| Alcohol (g) | 8.5 ± 13.6 | 0.0 ± 0.0 | 6.7 ± 19.3 | 6.8 ± 8.9 | 19.2 ± 21.6 | 0.0 ± 0.0 | 1.2 ± 2.5 | 16.1 ± 21.0* |
| Fibre (g) | 28.6 ± 7.2 | 7.0 ± 4.1 | 11.5 ± 6.6 | 10.8 ± 3.4 | 27.9 ± 7.9 | 6.5 ± 4.5 | 8.1 ± 6.1 | 12.6 ± 4.4 |

CHO, carbohydrate; TEI, total energy intake; TRF, time restricted feeding; URF, unrestricted feeding. Data are mean ± SD. Significantly (P<0.05) different to *TRF within time point.

*Qualitative questionnaire responses*

Broad opinions of TRF:

*No problems with the TRF pattern. I quite enjoyed it.* (Participant 2)

*I did generally feel better eating over a shorter window*. (Participant 3)

*I liked the restricted pattern. After getting used to a restricted pattern such as a late breakfast, it is fairly easy to stick to.* (Participant 10)

*I had initial difficulty with eating breakfast at 10am as I was hungry beforehand but I got used to this and it was ok.* (Participant 11)

*It is definitely possible for me to do and I will likely try it after this study* (Participant 8)

*I enjoyed eating meals in a shorter time frame as knew I couldn’t snack or eat anything in the evening so I didn’t*. *I wasn’t focused on snacking after dinner as much so it was a good thing for me.* (Participant 4)

*I liked the structured approach, which helped me plan work and home life.* (Participant 3)

*It was much easier to plan your day around your diet.* (Participant 4)

*The concept works well and anecdotally I could feel benefits like more energy* (Participant 3)

*I did generally find myself feeling better eating over a short window (*Participant 5)

Barriers towards a time-restricted feeding pattern:

*It is difficult with a 9 to 5 job as I would normally eat a lot in the evening when I get home as not much time in the day to eat.* (Participant 3)

*My current work schedule would be a huge barrier to implementing a regular 8-hour feeding pattern every day.* (Participant 11)

*Being too busy to eat particularly at work is a problem for me.* (Participant 2)

*Too much happening in the office means some days I don’t eat and if I can’t eat dinner late when I get home, I would starve.* (Participant 6)

*Socially it would be limiting.* (Participant 9)

*It would limit social outings to breakfast or dinner with family and friends.* (Participant 8)

*Social gatherings and night outs could be an issue.* (Participant 10)

*Restricting the food time frame would influence a lot of my normal social activities.* (Participant 3)

*In a family situation meal times are dependent on others so this is an issue.* (Participant 4)

*I missed out on our family dinners around the table.* (Participant 7)

*With kids at home, eating dinner before them or at the same time would lead to chaos.* (Participant 3)

*As a family person with kids, meal times work around a lot of other things like classes and schedules.* (Participant 5)

Perceived factors that make a time-restricted feeding pattern appealing:

*The structure and routine encourages you to get into good habits. You can make plans knowing whether you can or can’t eat.* (Participant 1)

*It helps you organize your workday*. (Participant 9)

*It seems a simple method to reduce your food intake without overcomplicating things.* (Participant 9)

*The ability to reduce the volume and calories of foods by just limiting the time window of intake is very appealing to me for weight loss.* (Participant 10)

*Potential for weight loss just by eating over less time seems too easy.* (Participant 11)

*It stops you eating a lot before bed, which I know it is not good to eat a lot before bed*. (Participant 1)

*It seems a simple way to limit my food intake.* (Participant 8)

*I actually liked the restricted patterns as it manages appetite and hunger and would likely limit the food I eat.* (Participant 10)

*The appealing thing for me is I was not snacking at all hours, which is my usual downfall* (Participant 2).

Feelings of hunger were not found to be a major barrier to a TRF eating pattern:

*I was hungry the first day, especially in the morning, and was eagerly waiting breakfast but this lessened each day.* (Participant 7)

*After getting used to the late breakfast it is fairly easy to stick to.* (Participant 10)

*I had initial difficulty with eating breakfast at 10am as I was hungry beforehand but I got used to this and it was ok.* (Participant 11)

*Going to bed hungry after the early dinner was difficult some days but not unbearable.* (Participant 2)

*I would get quite hungry in the evening but I got used to it so wasn’t worried.* (Participant 9)

*I didn’t feel very hungry when restricting time of intake*. (Participant 8)
